# Future Projection of Cystic Echinococcosis in Iran to 2040: A Secondary Analysis Based on Global Burden of Disease (2021)

**DOI:** 10.1002/hsr2.72725

**Published:** 2026-07-01

**Authors:** Behrooz Ahmadi, Meysam Olfatifar, Milad Badri, Mohammad Reza Ghadir

**Affiliations:** ^1^ Gastroenterology and Liver Diseases Research Center Research Institute for Gastroenterology and Liver Diseases, Shahid Beheshti University of Medical Sciences Tehran Iran; ^2^ Gastroenterology and Hepatology Diseases Research Center Qom University of Medical Sciences Qom Iran; ^3^ Medical Microbiology Research Center Qazvin University of Medical Sciences Qazvin Iran; ^4^ Student Research Committee Qazvin University of Medical Sciences Qazvin Iran

**Keywords:** age standardized prevalence rate (ASPR), *Echinococcus granulosus*, illness‐death model, Iran, projection, zoonotic disease

## Abstract

**Background and Aims:**

Hydatid cyst disease caused by *Echinococcus granulosus* poses a major zoonotic threat in Iran, especially in rural and pastoral areas. It causes considerable public health and economic costs due to high treatment expenses, decreased livestock productivity, and carcass condemnation.

**Methods:**

We applied the illness‐death model (IDM) by incorporating remission to simulate disease dynamics and project the age‐standardized prevalence rate (ASPR) by sex at the national level and across 31 provinces of Iran. The model was calibrated using historical data from the Global Burden of Disease (GBD).

**Results:**

Nationally, ASPR is projected to decline from 8.87 in 2021 to 7.3 (6.9–7.7) by 2040, reflecting a 17.7% reduction. Females experience a sharper decline (−25.4%) than males (−11.6%), despite higher initial prevalence. By 2040, Gilan, Isfahan, Lorestan, and Ilam will have the highest ASPRs, while Sistan and Baluchistan, Kermanshah, and South Khorasan will have the lowest. Provincial declines vary, with the steepest in Sistan and Baluchistan (−28.7%), Kermanshah (−27.1%), and South Khorasan (−26.7%); Isfahan, Gilan, and Lorestan will see the smallest declines.

**Conclusion:**

Iran has made notable progress in controlling CE, but geographic and sex‐based disparities persist due to socioecological and cultural differences. Success requires integrating zoonotic control with community‐driven approaches. By focusing on pastoral vaccination, urban dog management, and culturally tailored education, Iran can shift from reducing prevalence to achieving elimination.

## 1 Introduction

Cystic echinococcosis (CE), caused by the larval stage of the tapeworm *Echinococcus granulosus*, remains a significant public health burden in Iran, with profound implications for both human and animal populations [1]. This zoonotic infection thrives in a complex lifecycle involving dogs as definitive hosts and livestock, primarily sheep, goats, and cattle as intermediate hosts [2, 3]. Humans become accidental hosts through ingestion of parasite eggs in contaminated water or food, or via direct contact with infected animals [4, 5]. The disease's persistence in Iran is closely tied to pastoral and agricultural practices, where close interaction between livestock, dogs, and humans facilitates transmission. The most common clinical manifestations involve hepatic cysts (70% of cases) and pulmonary cysts (20%–30%), though cysts may also develop in other organs such as the brain, spleen, or kidneys [6, 7]. Untreated cases risk life‐threatening complications, including cyst rupture, secondary bacterial infections, or anaphylactic shock due to antigen release. Rural communities, particularly those engaged in livestock farming, face heightened exposure due to limited awareness and inadequate healthcare access.

The socioeconomic impact extends far beyond direct medical expenses. Annual economic losses in Iran exceed $130 million, driven largely by the culling of infected livestock, reduced meat and dairy production, and trade restrictions on contaminated animal products [8, 9, 10, 11]. Rural households, which rely heavily on livestock for income and subsistence, are disproportionately affected. Furthermore, the stigma associated with infected herds disrupts local markets and perpetuates cycles of poverty in endemic regions. Despite decades of control efforts, including dog deworming campaigns, meat inspection protocols, and public health education—Iran continues to report high infection rates. Key barriers include inconsistent enforcement of veterinary regulations, logistical challenges in delivering healthcare to remote areas, environmental contamination from stray dog populations, and cultural resistance to altering traditional farming practices [11, 12]. A crucial step in enhancing current strategies is to develop a robust modeling framework to pave the way for the future trajectory of CE. As these studies have pivotal roles in parasite epidemiology by providing better insights into disease management activities, they enable researchers and public health officials to predict disease trends, identify high‐risk areas, and evaluate the effectiveness of intervention measures.

The clinical and economic burden of CE remains substantial in Iran. An estimated 635,232 asymptomatic individuals live with the disease, annual costs surpass $232.3 million [9], and recent evidence reveals frequent deviation from WHO treatment guidelines in Iranian hospitals [13] along with ongoing diagnostic challenges in endemic areas [14]. To address these gaps, we developed an advanced illness‐death model (IDM) that incorporates remission rates, allowing for nuanced projections of disease trends. This model provides estimated projections at national and subnational levels across all 31 provinces of Iran, segmented by sex. It assists in making informed predictions to improve disease control by understanding its future trajectory.

## 2 Materials and Methods

### 2.1 Data Acquisition and Sources

This study integrates three primary data streams: the age‐standardized prevalence, incidence, and mortality rates of CE from 1990 to 2021, obtained by sex from the Global Burden of Disease (GBD) free access tools at https://vizhub.healthdata.org/gbd-results/. It should be noted that GBD estimates are themselves based on statistical modeling, particularly in settings with limited primary surveillance data; therefore, they carry inherent uncertainty that propagates into our projections. Additionally, population data for national and subnational levels, also separated by sex, were downloaded from the Statistical Centre of Iran (SCI) at https://amar.org.ir/.

### 2.2 Model Development and Calibration

In this study, we projected the future burden of CE in Iran using a discrete‐time IDM or susceptible–infected–susceptible (SIS) model structure. This framework is appropriate for CE since humans act as accidental dead‐end hosts; the model tracks the progression of human health states without explicitly simulating the full zoonotic transmission cycle, which is instead implicitly captured through the population‐level incidence rate. The model incorporates the following transitions: from Susceptible (S) to Infected (I); from Infected (I) back to Susceptible (S) upon successful treatment; from Infected (I) to Death (D) due to disease‐related mortality; and from Susceptible (S) to Death (D) resulting from non‐disease causes (see Figure S1).

The transitions are governed by parameters expressed as annual rates, each estimated using GBD data: the incidence rate represents the rate at which susceptible individuals (S) become infected (I) and was calibrated to match annual incidence data from the GBD study. The disease‐induced mortality rate, representing deaths directly attributable to CE, was derived from GBD cause‐specific mortality data. The background mortality rate, representing non‐CE deaths, was obtained from GBD all‐cause mortality records. The remission rate reflects the rate at which infected individuals (I) transition back to susceptible (S) following successful treatment. In this model, remission is treated as a transition event rather than a separate compartment, under the assumption that treatment clears infection without conferring lasting immunity, leaving individuals susceptible to reinfection. Due to the lack of direct remission data, this parameter was estimated using a discrete‐time formula based on GBD incidence, prevalence, and mortality data from 1990 to 2021 [15].

$$r_{k}=\frac{(1-n_{k}^{\left(1\right)})\omega_{k}-(1-n_{k}^{\left(0\right)})\omega_{k+1}+(1+\omega_{k+1})i_{k}}{(1-n_{k}^{\left(1\right)})\omega_{k}}$$

where *r_k_* is the remission rate at time *t*, *n_k_* ^(1)^ is the mortality risk among the diseased population at time *t*, *n_k_* ^(0)^ is the mortality risk among the susceptible population at time *t*, *ω_k_* is the prevalence odds of the disease at time *t_k_*, and *i_k_* is the incidence risk at time *t*. Model calibration utilized GBD data from 1990 to 2021, and goodness‐of‐fit was assessed via the root mean square error (RMSE) between model‐projected and GBD—age‐standardized prevalence rate (ASPR) during this period. Further details and source code are available from Ito et al. [15]. The model dynamics were simulated using a system of discrete‐time difference equations within an SIS framework as follows, incorporating sex‐specific parameters to account for differences in CE prevalence among females and occupational exposure risks among males.

$$S_{t+1}=-\theta_{01}S_{t}-\theta_{02}S_{t}+\theta_{10}I_{t}$$

$$I_{t+1}=+\theta_{01}S_{t}-\theta_{12}I_{t}-\theta_{10}I_{t}$$

where *S*_*t*_ and *I*_*t*_ represent the number of susceptible and infected individuals at time *t*, respectively; *θ*_01_ is the incidence rate (transition from *S* to *I*); *θ*_10_ is the remission rate (transition from *I* to *S*); and *θ*_02_ and *θ*_12_ represent the background (non‐disease) and disease‐specific mortality rates, respectively. This structure leverages the dynamic interaction between susceptible and infected populations to estimate recovery patterns, under the assumption that infected individuals either recover and re‐enter the susceptible pool or succumb to the disease. This approach enables a nuanced exploration of CE dynamics and supports the assessment of targeted interventions, with gender considered a key determinant of transmission and progression. By capturing interrelationships and temporal fluctuations among epidemiological rates, such as how incidence enlarges the infected compartment and how remission and mortality reduce it, the model offers a mechanistic basis for projecting future trends. Projections to 2040 were generated by running the calibrated system forward under the assumption that underlying rates of infection, treatment, and mortality remain consistent with the historical period, thus providing a baseline scenario of future CE burden.

### 2.3 Uncertainty Quantification and Additional Analysis

To address uncertainty in the GBD source data, all final estimates and projections are presented with 95% confidence intervals, derived from the model's parameter estimation process. Finally, to gain insights into broader epidemiological drivers, we evaluated the correlation between ASPR and the Human Development Index (HDI) at both national and subnational levels using the Pearson correlation test. HDI data were obtained from https://globaldatalab.org/shdi/. Briefly, this index is a number between −1 and +1, where −1 indicates a strong inverse relationship, and +1 indicates a strong synergistic relationship between the two variables. Moreover, we performed a Das Gupta decomposition analysis to quantify the contributions of changes in prevalence versus population dynamics to the total change in case numbers between 1990 and 2021.

### 2.4 Statistical Analysis

The statistical methods and reporting in this manuscript adhere to the principles of clinical research statistics [16]. All data processing, analysis, and visualization were conducted using R software (Version 4.3.1). Key packages utilized included splines for modeling, dplyr for data manipulation, ggplot2 for data visualization, and readxl, stringr, and fs for data management. We employed the Pearson correlation test to assess the relationship between ASPR and HDI. A priori significance levels were set at *p* < 0.05, and all tests were conducted as two‐sided. All analyses presented in this study, including national and subnational projections as well as subgroup analyses, were prespecified as part of the original study design.

## 3 Results

As noted in Section Methods, 2, all prevalence figures presented below are derived from GBD modeled estimates and should be interpreted with this in mind.

### 3.1 Historical and Future National Trends in CE Prevalence (1990–2040)

**Figure 2:**
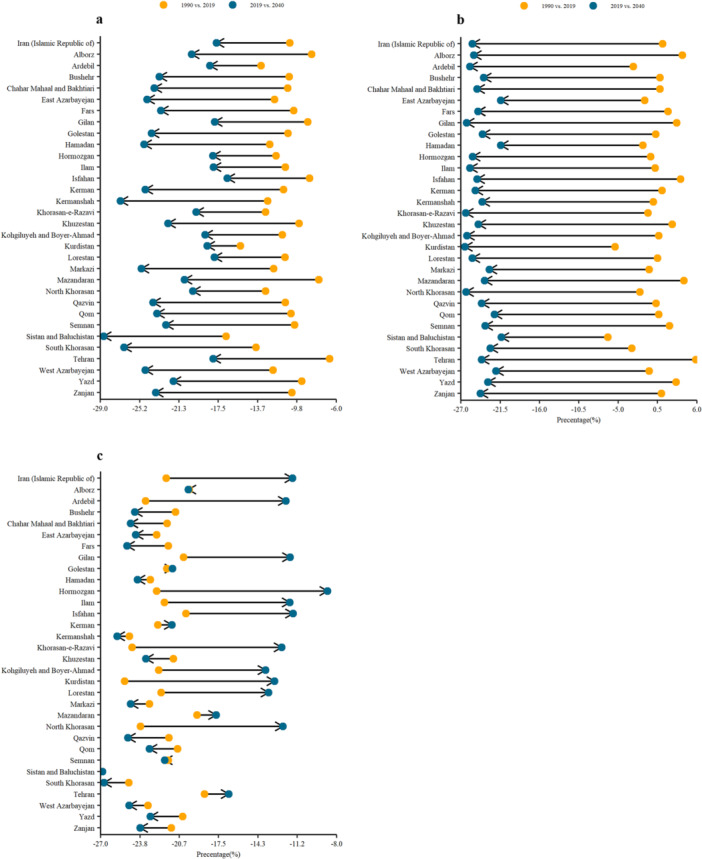
Percentage change in the age‐standardized prevalence rate (ASPR) of cystic echinococcosis (CE) across Iran and its 31 provinces. The plot compares two periods: the historical change from 1990 to 2021 (orange dots) and the projected change from 2021 to 2040 (blue dots). The arrows indicate the magnitude and direction of change between the two periods. Panels show data for (a) both sexes, (b) females, and (c) males.

The ASPR of CE at the national level is anticipated to decrease from 8.87 per 100,000 in 2021 to 7.30 per 100,000 (95% CI: 6.93–7.69) by 2040. This represents a reduction of 10.53% from 1990 to 2021 and 17.69% from 2021 to 2040 (Figure 2, Table 1). Among females, the ASPR is projected to decline from 9.80 per 100,000 in 2021 to 7.31 per 100,000 (95% CI: 7.25–7.36) by 2040, reflecting a 1.19% increase from 1990 to 2021, followed by a substantial 25.42% decrease from 2021 to 2040 (Figure 2, Table 2). In males, the ASPR is expected to decrease from 7.93 per 100,000 in 2021 to 7.02 per 100,000 (95% CI: 6.29–7.85) by 2040, corresponding to a reduction of 21.73% between 1990 and 2021 and 11.55% between 2021 and 2040 (Figure 1, Table 3).

**Table 1:** The estimated age‐standardized prevalence rates (ASPR) of cystic echinococcosis (CE) per 100,000 population for both sexes, along with 95% confidence intervals, are presented at 5‐year intervals from 2022 to 2040. It also records the percentage change in CE prevalence between 1990–2021 and 2021–2040 across Iran and its 31 provinces for both sexes, along with the root mean square error (RMSE).

	2022	2025	2030	2035	2040	Percentage change (1990–2021)	Percentage change (2021–2040)	RMSE
Iran (Islamic Republic of)	8.6 (8.5–8.8)	8.4 (8.2–8.6)	8 (7.8–8.3)	7.6 (7.3–8)	7.3 (6.9–7.7)	−10.5	−17.7	0.099
Alborz	8.7 (8.5–8.8)	8.4 (8.2–8.5)	7.9 (7.8–8.1)	7.5 (7.3–7.7)	7.1 (6.9–7.3)	−8.4	−20.1	0.100
Ardebil	8.7 (8.5–8.9)	8.5 (8.2–8.7)	8.1 (7.8–8.4)	7.7 (7.3–8)	7.3 (6.9–7.8)	−13.3	−18.3	0.099
Bushehr	8.5 (8.4–8.6)	8.2 (8.1–8.3)	7.7 (7.5–7.9)	7.2 (7.1–7.4)	6.8 (6.6–6.9)	−10.6	−23.3	0.098
Chahar Mahaal and Bakhtiari	8.6 (8.5–8.7)	8.3 (8.1–8.4)	7.8 (7.6–7.9)	7.3 (7.1–7.4)	6.8 (6.6–7)	−10.8	−23.8	0.102
East Azarbayejan	8.6 (8.4–8.7)	8.2 (8.1–8.4)	7.7 (7.6–7.9)	7.2 (7–7.4)	6.7 (6.6–6.9)	−12.0	−24.5	0.100
Fars	8.6 (8.4–8.7)	8.3 (8.1–8.4)	7.8 (7.6–7.9)	7.3 (7.1–7.4)	6.8 (6.7–7)	−10.2	−23.1	0.098
Gilan	8.8 (8.6–8.9)	8.5 (8.3–8.7)	8.1 (7.9–8.4)	7.8 (7.4–8.1)	7.4 (7–7.8)	−8.8	−17.9	0.099
Golestan	8.5 (8.4–8.7)	8.2 (8.1–8.4)	7.7 (7.5–7.9)	7.2 (7–7.4)	6.8 (6.6–6.9)	−10.7	−24.0	0.094
Hamadan	8.5 (8.4–8.7)	8.2 (8.1–8.4)	7.7 (7.5–7.8)	7.2 (7–7.3)	6.7 (6.5–6.8)	−12.5	−24.7	0.103
Hormozgan	8.5 (8.4–8.7)	8.3 (8.1–8.5)	7.9 (7.6–8.2)	7.5 (7.2–7.9)	7.2 (6.8–7.6)	−11.9	−18.0	0.098
Ilam	8.7 (8.5–8.9)	8.5 (8.3–8.7)	8.1 (7.8–8.3)	7.7 (7.4–8)	7.4 (7–7.7)	−11.0	−18.0	0.100
Isfahan	8.6 (8.5–8.8)	8.4 (8.2–8.6)	8.1 (7.8–8.3)	7.7 (7.4–8.1)	7.4 (7–7.8)	−8.6	−16.7	0.100
Kerman	8.5 (8.3–8.6)	8.1 (8–8.3)	7.6 (7.5–7.8)	7.1 (7–7.3)	6.6 (6.5–6.8)	−11.2	−24.6	0.099
Kermanshah	8.4 (8.3–8.5)	8.1 (7.9–8.2)	7.5 (7.3–7.6)	6.9 (6.8–7.1)	6.4 (6.3–6.6)	−12.7	−27.1	0.099
Khorasan‐e‐Razavi	8.5 (8.4–8.7)	8.3 (8.1–8.5)	7.8 (7.6–8.1)	7.5 (7.1–7.8)	7.1 (6.7–7.5)	−12.9	−19.7	0.098
Khuzestan	8.5 (8.4–8.7)	8.2 (8.1–8.4)	7.8 (7.6–7.9)	7.3 (7.1–7.5)	6.9 (6.7–7)	−9.6	−22.4	0.097
Kohgiluyeh and Boyer‐Ahmad	8.7 (8.5–8.8)	8.4 (8.2–8.6)	8 (7.8–8.3)	7.6 (7.3–8)	7.3 (6.9–7.7)	−11.3	−18.8	0.094
Kurdistan	8.7 (8.5–8.8)	8.4 (8.2–8.7)	8 (7.7–8.3)	7.6 (7.3–8)	7.3 (6.8–7.7)	−15.4	−18.6	0.103
Lorestan	8.7 (8.6–8.9)	8.5 (8.3–8.7)	8.1 (7.8–8.4)	7.7 (7.4–8.1)	7.4 (7–7.8)	−11.0	−17.9	0.101
Markazi	8.5 (8.4–8.6)	8.2 (8–8.3)	7.6 (7.5–7.8)	7.1 (7–7.3)	6.6 (6.5–6.8)	−12.1	−25.0	0.104
Mazandaran	8.7 (8.5–8.8)	8.4 (8.2–8.5)	7.9 (7.8–8.1)	7.5 (7.3–7.7)	7.1 (6.9–7.3)	−7.7	−20.8	0.099
North Khorasan	8.5 (8.4–8.7)	8.3 (8.1–8.5)	7.9 (7.6–8.1)	7.5 (7.2–7.8)	7.1 (6.7–7.5)	−12.9	−20.0	0.096
Qazvin	8.5 (8.4–8.6)	8.2 (8.1–8.3)	7.7 (7.5–7.8)	7.2 (7.1–7.3)	6.7 (6.6–6.9)	−11.0	−23.9	0.100
Qom	8.5 (8.4–8.6)	8.2 (8.1–8.3)	7.7 (7.5–7.8)	7.2 (7–7.3)	6.7 (6.6–6.9)	−10.4	−23.5	0.100
Semnan	8.5 (8.4–8.7)	8.2 (8.1–8.4)	7.7 (7.6–7.9)	7.3 (7.1–7.4)	6.8 (6.7–7)	−10.1	−22.6	0.102
Sistan and Baluchistan	8.2 (8.1–8.3)	7.8 (7.7–8)	7.2 (7.1–7.4)	6.7 (6.5–6.8)	6.2 (6–6.3)	−16.8	−28.7	0.095
South Khorasan	8.5 (8.4–8.6)	8.2 (8–8.3)	7.6 (7.4–7.7)	7 (6.9–7.2)	6.5 (6.4–6.7)	−13.8	−26.7	0.100
Tehran	8.6 (8.5–8.7)	8.4 (8.2–8.5)	8 (7.8–8.2)	7.6 (7.4–7.8)	7.3 (7–7.5)	−6.7	−18.0	0.100
West Azarbayejan	8.6 (8.5–8.7)	8.3 (8.1–8.4)	7.7 (7.6–7.9)	7.2 (7.1–7.4)	6.7 (6.6–6.9)	−12.2	−24.6	0.100
Yazd	8.6 (8.5–8.7)	8.3 (8.1–8.4)	7.8 (7.6–7.9)	7.3 (7.2–7.5)	6.9 (6.7–7.1)	−9.4	−21.9	0.096
Zanjan	8.6 (8.5–8.7)	8.3 (8.1–8.4)	7.8 (7.6–7.9)	7.3 (7.1–7.4)	6.8 (6.7–7)	−10.3	−23.6	0.100

**Table 2:** The estimated age‐standardized prevalence rates (ASPR) of cystic echinococcosis (CE) per 100,000 population for females, along with 95% confidence intervals, are presented at 5‐year intervals from 2022 to 2040. It also records the percentage change in CE prevalence between 1990–2021 and 2021–2040 across Iran and its 31 provinces for females, along with the root mean square error (RMSE).

	2022	2025	2030	2035	2040	Percentage change (1990–2021)	Percentage change (2021–2040)	RMSE
Iran (Islamic Republic of)	8.8 (8.8–8.8)	8.5 (8.5–8.5)	8.1 (8.1–8.1)	7.7 (7.6–7.7)	7.3 (7.3–7.4)	1.2	−25.4	0.112
Alborz	8.8 (8.8–8.8)	8.5 (8.5–8.6)	8.1 (8–8.2)	7.7 (7.6–7.8)	7.4 (7.3–7.5)	4.0	−25.2	0.116
Ardebil	8.9 (8.8–8.9)	8.6 (8.6–8.6)	8.1 (8.1–8.2)	7.7 (7.7–7.8)	7.3 (7.3–7.4)	−2.9	−25.7	0.108
Bushehr	8.8 (8.7–8.8)	8.5 (8.5–8.6)	8.1 (8–8.2)	7.8 (7.7–7.9)	7.4 (7.3–7.6)	0.8	−23.8	0.109
Chahar Mahaal and Bakhtiari	8.8 (8.8–8.8)	8.5 (8.5–8.6)	8.1 (8.1–8.2)	7.7 (7.7–7.8)	7.4 (7.4–7.4)	0.8	−24.7	0.114
East Azarbayejan	8.9 (8.8–8.9)	8.6 (8.6–8.7)	8.3 (8.3–8.3)	8 (8–8)	7.7 (7.7–7.7)	−1.3	−21.4	0.106
Fars	8.8 (8.8–8.8)	8.5 (8.5–8.6)	8.1 (8–8.2)	7.7 (7.6–7.8)	7.4 (7.3–7.5)	1.9	−24.6	0.111
Gilan	8.9 (8.8–8.9)	8.6 (8.6–8.6)	8.1 (8.1–8.2)	7.7 (7.7–7.8)	7.3 (7.3–7.4)	3.2	−26.2	0.116
Golestan	8.8 (8.8–8.8)	8.5 (8.5–8.6)	8.1 (8.1–8.2)	7.8 (7.7–7.8)	7.5 (7.4–7.5)	0.2	−24.0	0.101
Hamadan	8.9 (8.8–8.9)	8.6 (8.6–8.7)	8.3 (8.2–8.3)	8 (7.9–8)	7.7 (7.7–7.7)	−1.6	−21.4	0.112
Hormozgan	8.7 (8.7–8.7)	8.4 (8.4–8.5)	8 (8–8)	7.6 (7.6–7.7)	7.2 (7.2–7.3)	−0.5	−25.4	0.111
Ilam	8.8 (8.8–8.9)	8.6 (8.5–8.6)	8.1 (8.1–8.2)	7.7 (7.7–7.8)	7.3 (7.3–7.4)	0.2	−25.8	0.112
Isfahan	8.8 (8.8–8.8)	8.6 (8.5–8.6)	8.2 (8.1–8.2)	7.8 (7.7–7.8)	7.4 (7.3–7.5)	3.7	−24.7	0.112
Kerman	8.7 (8.7–8.7)	8.4 (8.4–8.5)	8 (8–8.1)	7.7 (7.6–7.7)	7.3 (7.3–7.4)	1.1	−25.0	0.113
Kermanshah	8.7 (8.7–8.7)	8.5 (8.4–8.5)	8.1 (8–8.1)	7.7 (7.7–7.8)	7.4 (7.4–7.5)	−0.1	−24.0	0.111
Khorasan‐e‐Razavi	8.7 (8.7–8.7)	8.4 (8.4–8.5)	8 (8–8)	7.6 (7.5–7.6)	7.2 (7.1–7.2)	−0.8	−26.3	0.111
Khuzestan	8.8 (8.7–8.8)	8.5 (8.5–8.5)	8.1 (8–8.1)	7.7 (7.6–7.8)	7.4 (7.3–7.5)	2.5	−24.6	0.114
Kohgiluyeh and Boyer‐Ahmad	8.8 (8.8–8.9)	8.6 (8.5–8.6)	8.1 (8.1–8.2)	7.7 (7.6–7.8)	7.3 (7.2–7.4)	0.6	−26.2	0.106
Kurdistan	8.8 (8.8–8.8)	8.5 (8.5–8.5)	8.1 (8–8.1)	7.6 (7.6–7.7)	7.2 (7.2–7.3)	−5.4	−26.5	0.115
Lorestan	8.8 (8.8–8.9)	8.6 (8.6–8.6)	8.2 (8.1–8.2)	7.8 (7.7–7.8)	7.4 (7.3–7.4)	0.5	−25.4	0.112
Markazi	8.8 (8.7–8.8)	8.5 (8.5–8.6)	8.2 (8.1–8.2)	7.8 (7.8–7.9)	7.5 (7.5–7.6)	−0.7	−23.0	0.114
Mazandaran	8.9 (8.9–8.9)	8.6 (8.6–8.6)	8.2 (8.2–8.2)	7.9 (7.8–7.9)	7.5 (7.5–7.6)	4.2	−23.7	0.109
North Khorasan	8.7 (8.7–8.8)	8.5 (8.4–8.5)	8 (8–8.1)	7.6 (7.6–7.6)	7.2 (7.2–7.3)	−2.0	−26.3	0.105
Qazvin	8.7 (8.7–8.8)	8.5 (8.4–8.5)	8.1 (8–8.1)	7.7 (7.7–7.8)	7.4 (7.4–7.5)	0.3	−24.1	0.113
Qom	8.8 (8.8–8.8)	8.6 (8.5–8.6)	8.2 (8.2–8.2)	7.9 (7.9–7.9)	7.6 (7.6–7.6)	0.6	−22.3	0.110
Semnan	8.8 (8.8–8.8)	8.5 (8.5–8.6)	8.1 (8.1–8.2)	7.8 (7.7–7.9)	7.5 (7.3–7.6)	2.2	−23.6	0.108
Sistan and Baluchistan	8.6 (8.6–8.7)	8.4 (8.4–8.4)	8.1 (8–8.1)	7.8 (7.7–7.8)	7.5 (7.5–7.6)	−6.5	−21.4	0.100
South Khorasan	8.8 (8.8–8.8)	8.6 (8.5–8.6)	8.2 (8.1–8.2)	7.8 (7.8–7.9)	7.6 (7.6–7.6)	−3.1	−22.9	0.107
Tehran	8.8 (8.7–8.8)	8.5 (8.5–8.5)	8.1 (8.1–8.1)	7.8 (7.7–7.8)	7.4 (7.4–7.4)	5.8	−24.1	0.114
West Azarbayejan	8.9 (8.9–8.9)	8.7 (8.6–8.7)	8.3 (8.3–8.3)	8 (8–8)	7.7 (7.7–7.7)	−0.7	−22.1	0.113
Yazd	8.8 (8.8–8.8)	8.6 (8.6–8.6)	8.2 (8.2–8.2)	7.8 (7.8–7.9)	7.5 (7.5–7.5)	3.1	−23.2	0.106
Zanjan	8.8 (8.8–8.9)	8.6 (8.5–8.6)	8.2 (8.1–8.2)	7.8 (7.7–7.9)	7.5 (7.3–7.6)	1.0	−24.3	0.112

Figure 1The historical (1990–2021) and projected (2021–2040) age‐standardized prevalence rates (ASPR) of cystic echinococcosis (CE) per 100,000 individuals across the 31 provinces of Iran. Data are presented for both sexes combined (red line), females (green line), and males (blue line). The shaded area (halo effect) surrounding the projection lines represents the 95% confidence interval for the estimated values.

**Figure:**
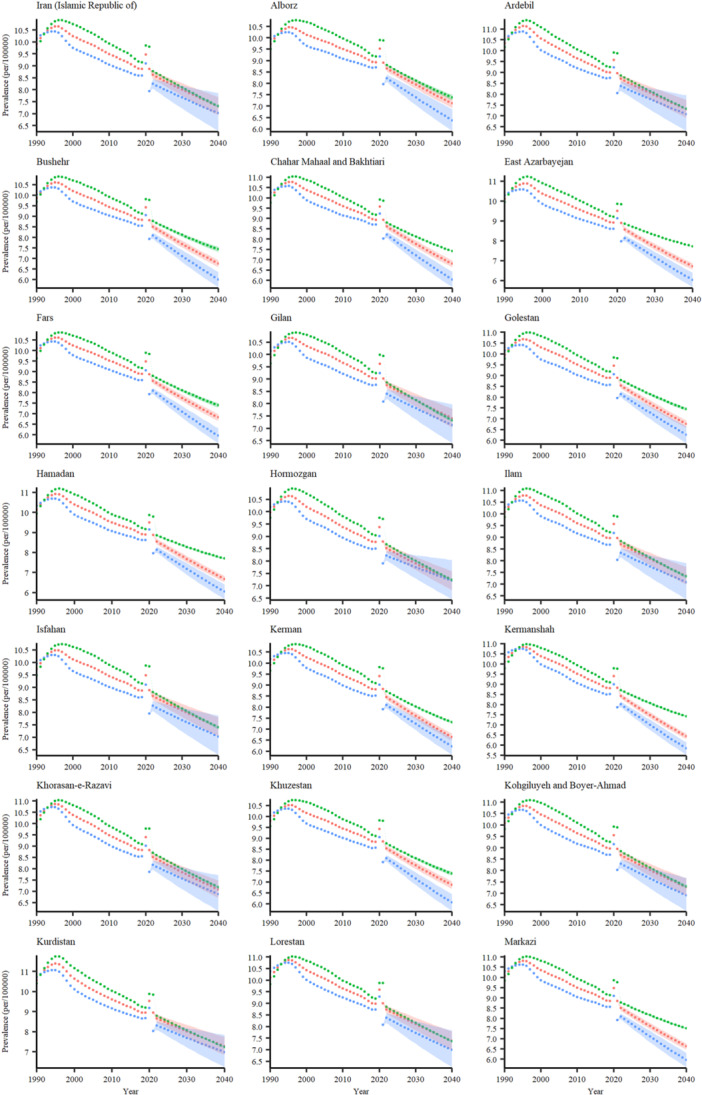

**Figure:**
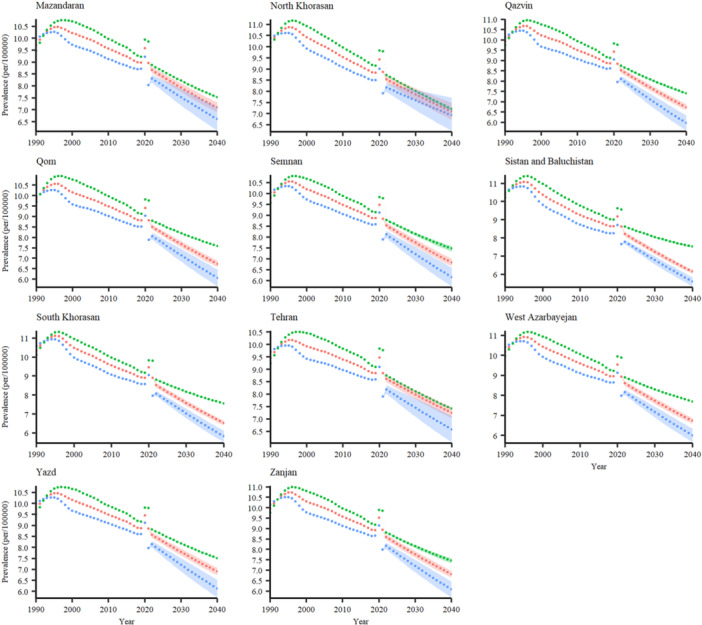

**Table 3:** The estimated age‐standardized prevalence rates (ASPR) of cystic echinococcosis (CE) per 100,000 population for males, along with 95% confidence intervals, are presented at 5‐year intervals from 2022 to 2040. It also records the percentage change in CE prevalence between 1990–2021 and 2021–2040 across Iran and its 31 provinces for males, along with the root mean square error (RMSE).

	2022	2025	2030	2035	2040	Percentage change (1990–2021)	Percentage change (2021–2040)	RMSE
Iran (Islamic Republic of)	8.2 (8.1–8.5)	8 (7.7–8.3)	7.7 (7.2–8.2)	7.3 (6.7–8)	7 (6.3–7.9)	−21.7	−11.6	0.142
Alborz	8.2 (8.1–8.4)	7.9 (7.7–8.1)	7.4 (7–7.7)	6.9 (6.5–7.3)	6.4 (5.9–6.9)	−19.9	−20.0	0.146
Ardebil	8.4 (8.2–8.6)	8.1 (7.8–8.5)	7.8 (7.3–8.3)	7.4 (6.8–8.1)	7.1 (6.3–8)	−23.4	−12.1	0.147
Bushehr	8.1 (7.9–8.2)	7.7 (7.5–7.9)	7.1 (6.8–7.4)	6.6 (6.2–6.9)	6 (5.7–6.4)	−21.0	−24.3	0.135
Chahar Mahaal and Bakhtiari	8.2 (8.1–8.4)	7.8 (7.6–8)	7.2 (6.9–7.5)	6.6 (6.3–7)	6.1 (5.7–6.4)	−21.7	−24.6	0.144
East Azarbayejan	8.1 (8–8.3)	7.8 (7.6–8)	7.2 (6.9–7.5)	6.6 (6.3–6.9)	6 (5.7–6.4)	−22.5	−24.2	0.142
Fars	8.1 (8–8.2)	7.7 (7.5–7.9)	7.1 (6.8–7.4)	6.5 (6.2–6.8)	6 (5.6–6.3)	−21.6	−24.9	0.136
Gilan	8.4 (8.2–8.6)	8.2 (7.9–8.5)	7.8 (7.4–8.3)	7.5 (6.9–8.1)	7.1 (6.4–8)	−20.3	−11.8	0.141
Golestan	8.1 (8–8.3)	7.8 (7.6–8)	7.3 (7–7.6)	6.8 (6.4–7.1)	6.3 (5.9–6.6)	−21.7	−21.2	0.137
Hamadan	8.1 (8–8.3)	7.8 (7.6–8)	7.2 (6.9–7.5)	6.6 (6.3–6.9)	6 (5.7–6.4)	−23.0	−24.0	0.145
Hormozgan	8.2 (8–8.4)	8 (7.7–8.3)	7.8 (7.3–8.2)	7.5 (6.9–8.1)	7.2 (6.5–8.1)	−22.5	−8.7	0.138
Ilam	8.3 (8.1–8.5)	8.1 (7.8–8.4)	7.8 (7.3–8.2)	7.4 (6.8–8.1)	7.1 (6.4–7.9)	−21.9	−11.8	0.140
Isfahan	8.3 (8.1–8.5)	8 (7.7–8.4)	7.7 (7.2–8.2)	7.4 (6.8–8)	7 (6.3–7.9)	−20.1	−11.5	0.141
Kerman	8.1 (8–8.2)	7.8 (7.6–8)	7.2 (7–7.5)	6.7 (6.4–7.1)	6.2 (5.9–6.6)	−22.4	−21.3	0.140
Kermanshah	8 (7.9–8.1)	7.6 (7.4–7.8)	7 (6.8–7.3)	6.4 (6.1–6.7)	5.8 (5.5–6.1)	−24.7	−25.7	0.143
Khorasan‐e‐Razavi	8.2 (8–8.4)	7.9 (7.6–8.2)	7.6 (7.1–8.1)	7.2 (6.6–7.9)	6.9 (6.2–7.7)	−24.5	−12.4	0.143
Khuzestan	8.1 (7.9–8.2)	7.7 (7.5–7.9)	7.2 (6.9–7.4)	6.6 (6.3–6.9)	6.1 (5.7–6.4)	−21.1	−23.4	0.137
Kohgiluyeh and Boyer‐Ahmad	8.3 (8.1–8.5)	8.1 (7.8–8.3)	7.7 (7.2–8.1)	7.3 (6.7–7.9)	6.9 (6.2–7.7)	−22.3	−13.7	0.135
Kurdistan	8.3 (8.1–8.5)	8.1 (7.8–8.4)	7.7 (7.2–8.2)	7.3 (6.7–8)	7 (6.2–7.8)	−25.1	−13.0	0.142
Lorestan	8.4 (8.2–8.6)	8.1 (7.8–8.4)	7.7 (7.3–8.2)	7.4 (6.7–8)	7 (6.3–7.8)	−22.1	−13.5	0.145
Markazi	8.1 (8–8.2)	7.7 (7.5–7.9)	7.1 (6.8–7.4)	6.5 (6.2–6.9)	6 (5.6–6.3)	−23.1	−24.6	0.142
Mazandaran	8.3 (8.1–8.5)	8 (7.8–8.3)	7.5 (7.2–7.9)	7.1 (6.6–7.5)	6.6 (6.1–7.1)	−19.2	−17.7	0.144
North Khorasan	8.2 (8–8.4)	8 (7.7–8.3)	7.6 (7.2–8.1)	7.3 (6.7–7.9)	6.9 (6.2–7.7)	−23.8	−12.3	0.135
Qazvin	8.1 (8–8.2)	7.7 (7.5–7.9)	7.1 (6.8–7.4)	6.5 (6.2–6.9)	6 (5.6–6.3)	−21.5	−24.8	0.140
Qom	8.1 (7.9–8.2)	7.7 (7.5–7.9)	7.1 (6.8–7.4)	6.6 (6.2–7)	6.1 (5.7–6.5)	−20.8	−23.1	0.142
Semnan	8.1 (8–8.3)	7.8 (7.5–8)	7.2 (6.9–7.5)	6.7 (6.3–7.1)	6.2 (5.7–6.6)	−21.6	−21.8	0.148
Sistan and Baluchistan	7.8 (7.7–7.9)	7.4 (7.2–7.6)	6.8 (6.6–7)	6.2 (5.9–6.4)	5.6 (5.4–5.8)	−27.0	−26.9	0.135
South Khorasan	8.1 (7.9–8.2)	7.7 (7.5–7.9)	7 (6.8–7.3)	6.4 (6.1–6.7)	5.8 (5.5–6.1)	−24.7	−26.7	0.142
Tehran	8.2 (8–8.4)	7.9 (7.7–8.2)	7.4 (7.1–7.8)	7 (6.6–7.5)	6.6 (6.1–7.1)	−18.6	−16.7	0.147
West Azarbayejan	8.2 (8–8.3)	7.8 (7.6–8)	7.2 (6.9–7.4)	6.6 (6.3–6.9)	6 (5.7–6.4)	−23.2	−24.7	0.142
Yazd	8.1 (8–8.3)	7.8 (7.6–8)	7.2 (6.9–7.5)	6.7 (6.3–7)	6.1 (5.7–6.5)	−20.4	−23.0	0.139
Zanjan	8.2 (8–8.3)	7.8 (7.6–8)	7.2 (6.9–7.5)	6.6 (6.3–7)	6.1 (5.7–6.5)	−21.3	−23.8	0.142

### 3.2 Historical and Future Subnational Trends in CE Prevalence (1990–2040)

**Figure 3:**
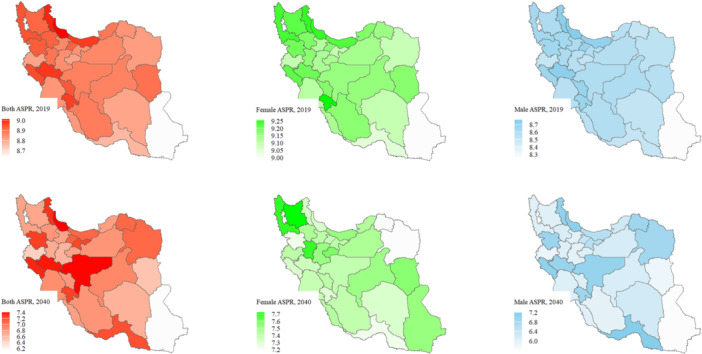
Geographical distribution of the age‐standardized prevalence rates (ASPRs) of cystic echinococcosis (CE) across the 31 provinces of Iran. The top row of maps displays the estimated ASPR in 2021, while the bottom row shows the projected ASPR for 2040. Maps are presented for both sexes combined (red color scale), females (green color scale), and males (blue color scale). For each map, darker shades of color indicate a higher disease burden (higher ASPR).

Substantial geographic variation in CE burden was observed. In 2021, the highest ASPRs were concentrated in the western and northwestern provinces for both sexes, with Gilan, Lorestan, and Ilam representing the highest‐burden areas. By 2040, while all provinces are projected to see a decline, this geographic pattern is expected to persist. Gilan, Isfahan, Lorestan, and Ilam are projected to remain the highest‐burden provinces (ASPR > 7.35 per 100,000), albeit with significantly lower absolute values. Conversely, the southeastern provinces consistently demonstrated the lowest burden. Sistan and Baluchistan, Hormozgan, and Qom had the lowest ASPRs in 2021. By 2040, Sistan and Baluchistan, Kermanshah, and South Khorasan are projected to have the lowest values (ASPR < 6.55 per 100,000). The magnitude of the projected decline also varied geographically. The greatest percentage reductions are anticipated in the southeastern and western regions (Sistan and Baluchistan: −28.7%, Kermanshah: −27.1%, and South Khorasan: −26.7%). In contrast, the smallest reductions were projected for the central and high‐burden western provinces (Isfahan: −16.7%, Gilan: −17.9%, and Lorestan: −17.9%), suggesting a potential narrowing of relative disparities over time (Figures 1, 2, 3, Table 1).

Similarly, the highest female ASPRs in 2021 were concentrated in northwestern and western regions, with Gilan, West Azarbayejan, and Kohgiluyeh and Boyer‐Ahmad representing the highest‐burden areas. By 2040, while all provinces are projected to experience declines, the spatial distribution of high‐burden areas is expected to shift eastward. East Azarbayejan, West Azarbayejan, and Hamadan are projected to emerge as the highest‐burden provinces (ASPR > 7.69 per 100,000), despite overall lower absolute values. Conversely, southeastern and central provinces consistently showed the lowest disease burden among females. Sistan and Baluchistan, Hormozgan, and Markazi recorded the lowest ASPRs in 2021.

By 2040, Khorasan‐e‐Razavi, North Khorasan, and Kurdistan are projected to have the lowest ASPR values (ASPR < 7.25 per 100,000). The projected rate of improvement also demonstrated geographic heterogeneity. The most substantial percentage reductions are anticipated in northwestern and northeastern regions (Kurdistan: −26.5%, Khorasan‐e‐Razavi: −26.3%, and North Khorasan: −26.3%). In contrast, the most modest declines were projected for southeastern and eastern provinces (Sistan and Baluchistan: −21.4%, East Azarbayejan: −21.4%, and Hamadan: −21.4%), indicating varying paces of epidemiological transition across different geographic regions (Figures 1, 2, 3, Table 2).

Among males, the highest disease prevalence in 2021 was found predominantly in northern and western provinces, particularly in Gilan, Lorestan, and Ardabil. Projections for 2040 indicate a notable geographical shift in disease burden toward southern regions, with Hormozgan expected to show the highest ASPR (7.20 per 100,000), followed by Gilan (7.13), and both Ardebil and Ilam (7.08). Eastern provinces consistently showed the lowest disease burden, with Sistan and Baluchistan, Khorasan‐e‐Razavi, and Kermanshah recording the lowest ASPR values in 2021.

By 2040, Sistan and Baluchistan is projected to maintain the lowest prevalence (5.60 per 100,000), accompanied by South Khorasan (5.82) and Kermanshah (5.83). The projected epidemiological transition shows considerable regional variation, with the most substantial percentage reductions expected in eastern provinces (Sistan and Baluchistan: −26.9%, South Khorasan: −26.7%, Kermanshah: −25.7%). Conversely, the most modest improvements are forecast for southern and central provinces (Hormozgan: −8.7%, Isfahan: −11.5%, Gilan: −11.8%, Ilam: −11.8%), indicating significant regional differences in the pace of epidemiological transition (Figures 1, 2, 3, Table 3).

### 3.3 Correlation and Decomposition Analysis

**Figure 4:**
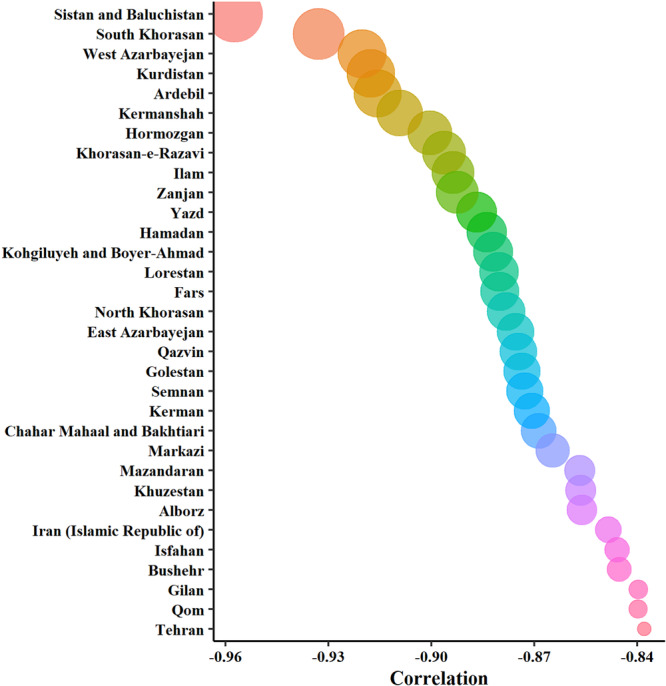
Relationship between provincial health outcomes and socioeconomic development in Iran (1990–2021). Each bubble represents a province, with its position determined by the average age‐standardized prevalence rate (ASPR) of cystic echinococcosis (CE) and its association with the Human Development Index (HDI) over the period. The size of the bubble indicates the strength of the temporal correlation between the province's annual ASPR and annual HDI across the 32‐year period, with larger bubbles representing a stronger correlation.

**Figure 5:**
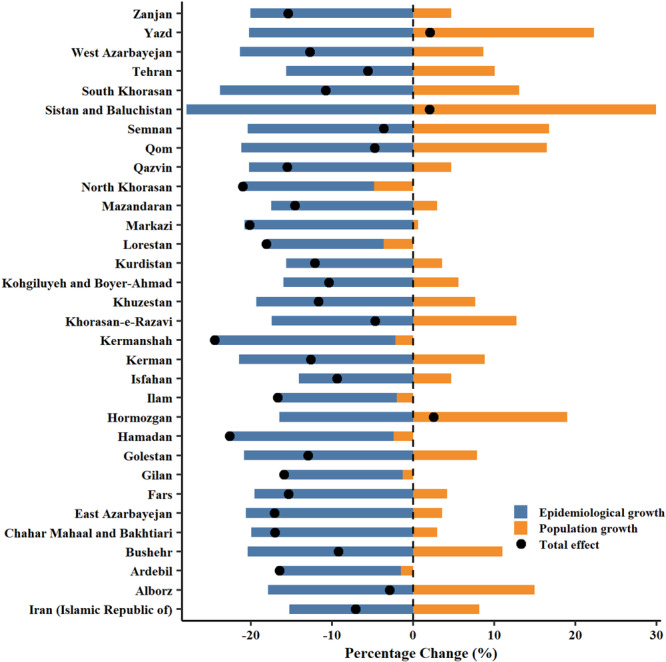
Results of the Das Gupta decomposition analysis for the change in cystic echinococcosis (CE) burden from 2021 to 2040. The bars represent the total change in CE cases for each province, which is decomposed into two components: the portion attributable to demographic shifts (population growth) and the portion attributable to epidemiological changes (changes in the age‐standardized prevalence rate, ASPR). The dot point represents the total effect.

At the national and provincial levels, we observed a negative correlation between ASPRs for both sexes and HDI: lower ASPRs were associated with higher HDI values, and vice versa (Figure 4). Sistan and Baluchestan experienced the largest ASPR decline from 1990 to 2021 (16.76), while Tehran and Mazandaran saw the smallest declines (Table 1 and Figure 2), maintaining their relative positions in Figure 4. Morover, based on the decomposition analysis, the observed changes in CE burden across Iranian provinces between 1990 and 2021 were primarily driven by epidemiological factors rather than demographic shifts. The analysis revealed a pronounced overall decline in CE prevalence, largely attributable to reductions in transmission and improvements in treatment efficacy, reflecting the success of past public health efforts. However, considerable heterogeneity was observed at the provincial level, with some regions experiencing more substantial reductions than others (Figure 5). This underscores the importance of targeted, subnational interventions to address lingering hotspots and ensure continued progress toward national control targets.

### 3.4 Model Calibration

The model demonstrated a strong fit to the historical data across all provinces and demographic groups. The RMSE values were consistently low at the national level (0.099 for both sexes combined, 0.112 for females, and 0.142 for males). This pattern of strong performance was maintained across all 31 provinces, with provincial RMSE values for the total population ranging from 0.094 to 0.104 (Tables 1, 2, 3).

## 4 Discussion

Our study offers valuable insights into the future trajectory of CE for health policymakers to consider in formulating effective control strategies. Despite ongoing control efforts, CE continues to pose a significant public health challenge in Iran, exacerbated by inequities driven by zoonotic reservoirs, occupational hazards, and disparities in healthcare access.

The projected decline reflects the effectiveness of integrated control strategies, including dog deworming, livestock vaccination, and public health education campaigns targeting rural populations, where the CE burden is most pronounced [17, 18]. This result is consistent with some reports in Turkana and Kenya [19]. Nevertheless, Iran's progress remains susceptible to setbacks due to persistent zoonotic reservoirs, compounded by clinical mismanagement such as unnecessary surgery for inactive cysts as documented in northeastern Iran [13]. The prevalence of CE in livestock remains alarmingly high, as evidenced by a recent meta‐analysis indicating a relatively high prevalence of CE in slaughtered livestock, including sheep, goats, cows, buffalo, and camels, at 13.9%, with the highest prevalence observed in camels [20].

This situation perpetuates transmission cycles despite interventions focused on human populations. Because the prevalence of CE in humans is closely correlated with and dependent on the prevalence of CE in livestock, as opposed to canines. This relationship is evidenced by the geographical distribution of CE in human and animal hosts in Europe, where the pattern observed in humans aligns with that of livestock [21]. This observation aligns with research findings from Argentina, where the implementation of livestock vaccination programs significantly reduced disease prevalence. Consequently, there was an approximate 90% decrease in human prevalence, from 5.6% in 2009 to 0.12% in 2015. This highlights the critical importance of continuous veterinary efforts [22, 23, 24].

On the other hand, one evidence from Italy indicates that a One Health approach, characterized by multidisciplinary and multi‐institutional collaboration, is essential for the effective control of CE. Therefore, it is imperative to rigorously adhere to current strategies and implement targeted interventions in collaboration with institutions beyond the health sector to progress toward elimination [25]. Furthermore, the findings of this study underscore the necessity for long‐term programs aimed at controlling and eliminating CE. An 8‐year integrated program in southern Italy resulted in a reduction of the infection rate in sheep by up to 30%. Similar outcomes have been observed in South America following the implementation of One Health approaches [26]. Therefore, despite the lack of complete control over CE in this region, the long‐term activities have yielded positive results. Furthermore, the provincial disparities in CE reduction, with the steepest declines in southeastern and western provinces and the smallest in certain central and high‐burden western provinces, highlight the critical role of socioecological and cultural factors in shaping transmission dynamics. Some interconnected drivers likely explain these inequities: First, the prevalence of extensive pastoralism in specific provinces, such as Kermanshah, Ilam, Lorestan, and Gilan [27], and subsequently the extent of livestock vaccination in these regions are noteworthy. Second, there are regional variations in the prevalence of infected stray dogs, with 50% in Tehran, 22% in Khorasan‐e‐Razavi, 27.1% in Isfahan, and 9% in Ilam [28, 29, 30, 31].

These variations can be directly correlated with the burden of CE, reflecting disparities in dog population management. Additionally, the differing levels of health education among communities and the practice of feeding raw viscera to dogs, which perpetuates the parasite cycle, are crucial considerations [32]. It seems that the latter practice has become more widespread in provinces such as Tehran and Isfahan, attributed to the growing trend of dog walking and keeping dogs as pets rather than as guard dogs in these areas. This is supported by the recent emergence of online stores supplying raw animal food in Iran (https://rawfeedingiran.com/). An in‐depth analysis of the correlation between ASPRs of CE and HDI (Figure 4) reveals that this index alone is insufficient to predict the epidemiological distribution of CE across the nation. For example, Sistan and Baluchistan, despite possessing a lower HDI than Tehran, have experienced a more substantial reduction in ASPR from 1990 to 2021, indicating a stronger correlation between these variables.

This phenomenon may be attributed to previously discussed factors, suggesting that Tehran's high HDI may obscure unaddressed urban risks, such as pet‐related cultural practices. Consequently, the uniform implementation of elimination programs across Iran's provinces can be misleading without considering socio‐cultural factors. Evidence indicates these factors are frequently overlooked in control programs due to challenges in population persuasion [24]. To effectively eliminate CE, integrated, comprehensive nationwide efforts are essential, supported by a robust surveillance system that accounts for socio‐cultural dynamics, as evidenced by provincial examples [33]. Moreover, it is imperative to meticulously examine the discrepancies between our findings and those of other studies, such as the one conducted in Kermanshah [34], which indicated an increasing trend of CE in this province. This discrepancy arises from the use of ASPR rather than solely relying on raw prevalence rates, thereby facilitating comparisons between provinces by neutralizing the population pattern based on age. As reported by GBD, at https://vizhub.healthdata.org/gbd-results/, the non‐standardized prevalence of CE for this province has shown an increasing trend from 1990 to 2021.

A significant aspect to consider is the more pronounced decline in the female ASPR by 2040. This trend may partly be attributable to enhanced healthcare access and hygiene initiatives targeting rural women, who traditionally oversee food preparation and are disproportionately exposed to contaminated water and vegetables. However, higher healthcare utilization among women could also contribute to this pattern. Studies have documented greater outpatient service use by women in Iran [35], and Iranian mothers receive an average of 5.9 ultrasound scans during pregnancy [36], which may increase incidental detection of hepatic cysts and subsequent treatment. Conversely, the slower progress in males may reflect persistent occupational hazards, particularly among herders and slaughterhouse workers. These trends support the necessity for gender‐sensitive interventions. For instance, while hygiene programs focused on women have been effective in reducing fecal‐oral transmission, occupational safety measures for males, such as the distribution of protective gear and regular health screenings, remain underemphasized.

Although this is the first study to model the future epidemiology of CE in Iran using an enhanced IDM framework, our analysis has several limitations. Primarily, our projections rely on the GBD 2021 database, which, as a modeled estimate itself, may not fully capture the true situation in rural or underserved provinces where surveillance is limited. To explicitly account for this uncertainty, we have presented all our estimates, including the future prevalence projections with 95% confidence intervals derived from our model. These intervals represent the range of plausible outcomes given the uncertainties in the underlying data.

Therefore, our results should be interpreted as a “best‐estimate” baseline within a quantified range of uncertainty, rather than a definitive prediction. In the absence of a comprehensive, nationwide surveillance system, the GBD remains the most robust available source for such an analysis, and this study establishes a critical foundation for further research. It offers the insights necessary for the swift implementation and targeted refinement of elimination programs nationwide. In summary, to effectively eliminate CE, Iran should adopt a decentralized and context‐specific approach. This strategy should encompass enhancing the vaccination rates among livestock, dispatching veterinary teams to remote regions for dog deworming and educational initiatives, establishing robust digital registries for monitoring and sterilizing stray dogs, banning the commercial distribution of raw viscera for pet consumption, creating a One Health surveillance system that integrates data from human, animal, and environmental sources, and equipping local leaders in high‐risk areas to conduct educational campaigns.

## 5 Conclusion

Iran's CE decline is a testament to sustained public health efforts, but geographic and sex‐based disparities persist due to socioecological and cultural differences. A one‐size‐fits‐all approach will fail; success hinges on integrating zoonotic control with community‐driven solutions. By prioritizing pastoral vaccination, urban dog management, and culturally adapted education, Iran can transition from prevalence reduction to elimination. Moreover, elimination requires decentralized strategies prioritizing zoonotic control, gender‐sensitive interventions, and robust surveillance. This study provides a roadmap for tailoring policies to local socioecological contexts, critical for achieving national and global CE elimination targets.

## Author Contributions

**Behrooz Ahmadi:** conceptualization, writing – review and editing, writing – original draft. **Meysam Olfatifar:** conceptualization, methodology, software, formal analysis, writing – original draft, writing – review and editing, funding acquisition, project administration, supervision, visualization. **Milad Badri:** conceptualization, writing – review and editing, writing – original draft. **Mohammad Reza Ghadir:** conceptualization, writing – review and editing, writing – original draft.

## Ethics Statement

This project was approved by the Ethics Committee of Qom University of Medical Sciences (IR.MUQ.REC.1403.288).

## Consent

The authors have nothing to report.

## Conflicts of Interest

The authors declare no conflicts of interest.

## Transparency Statement

Meysam Olfatifar affirms that this manuscript is an honest, accurate, and transparent account of the study being reported; that no important aspects of the study have been omitted; and that any discrepancies from the study as planned (and, if relevant, registered) have been explained.

## Supporting information


**Figure S1:** Schematic representation of illness death model (IDM).

## Data Availability

The GBD data used in this study can be accessed freely at https://vizhub.healthdata.org/gbd-results/ by selecting “Cystic echinococcosis” as the cause, “Iran (Islamic Republic of)” as the location, and “Age‐standardized prevalence/incidence/mortality rate” as the measure. Population data were obtained from https://amar.org.ir/. The corresponding analysis codes are available at https://zenodo.org/records/6379226.
