# Supplementary figures and images for: Future Projection of Cystic Echinococcosis in Iran to 2040: A Secondary Analysis Based on Global Burden of Disease (2021)
Source: Health Sci Rep. 2026 Jul 1;9(7):e72725. doi: 10.1002/hsr2.72725 (PMC13323838; doi:10.1002/hsr2.72725)

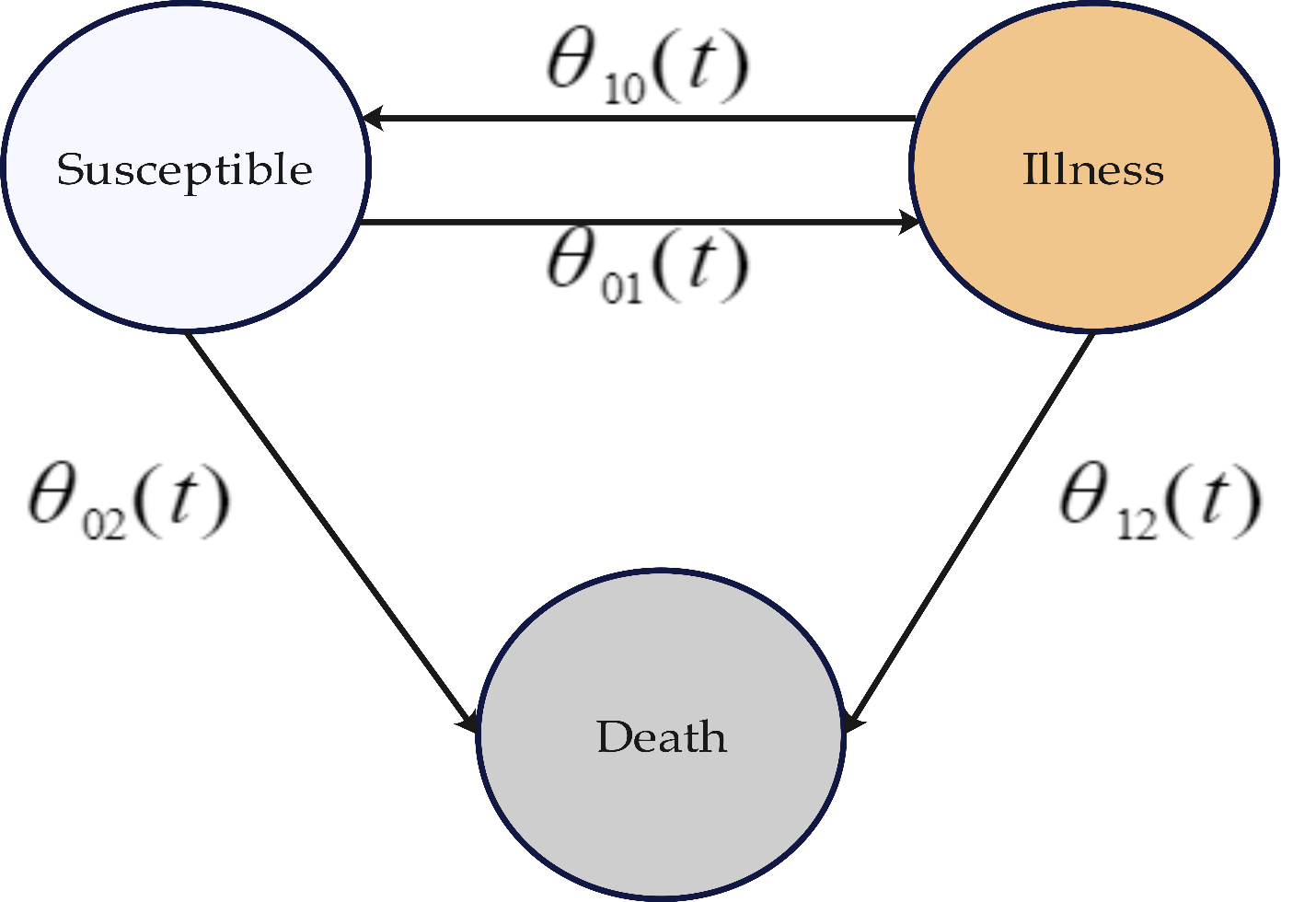


Figure 1: Schematic representation of illness death model (IDM)

Supplement: Supplementary file 1 — Figure S1: Schematic representation of illness death model (IDM). [file HSR2-9-e72725-s001.docx]
